# DNA Methylation Is Involved in the Expression of miR-142-3p in Fibroblasts and Induced Pluripotent Stem Cells

**DOI:** 10.1155/2014/101349

**Published:** 2014-12-02

**Authors:** Siti Razila Abdul Razak, Yukihiro Baba, Hiromitsu Nakauchi, Makoto Otsu, Sumiko Watanabe

**Affiliations:** ^1^Division of Molecular and Developmental Biology, Institute of Medical Science, University of Tokyo, 4-6-1 Shirokanedai, Minato-ku, Tokyo 108-8639, Japan; ^2^Division of Stem Cell Therapy, Center for Stem Cell Biology and Regenerative Medicine, Institute of Medical Science, University of Tokyo, 4-6-1 Shirokanedai, Minato-ku, Tokyo 108-8639, Japan

## Abstract

MicroRNAs are differentially expressed in cells and regulate multiple biological processes. We have been analyzing comprehensive expression patterns of microRNA in human and mouse embryonic stem and induced pluripotent stem cells. We determined microRNAs specifically expressed in these pluripotent stem cells, and miR-142-3p is one of such microRNAs. miR-142-3p is expressed at higher levels in induced pluripotent stem cells relative to fibroblasts in mice. Level of expression of miR142-3p decreased during embryoid body formation from induced pluripotent stem cells. Loss-of-function analyses of miR-142-3p suggested that miR-142-3p plays roles in the proliferation and differentiation of induced pluripotent stem cells. CpG motifs were found in the 5′ genomic region of the *miR-142-3p*; they were highly methylated in fibroblasts, but not in undifferentiated induced pluripotent stem cells. Treating fibroblasts with 5-aza-2′-deoxycytidine increased the expression of miR-142-3p significantly and reduced methylation at the CpG sites, suggesting that the expression of miR-142-3p is suppressed by DNA methylation in fibroblasts. Luciferase analysis using various lengths of the 5′ genomic region of miR142-3p indicated that CpGs in the proximal enhancer region may play roles in suppressing the expression of miR-142-3p in fibroblasts.

## 1. Introduction

The self-renewal and differentiation of pluripotent stem cells are regulated by various factors including growth factors, cytokines, intracellular signaling molecules, the extracellular matrix, and transcription factors. In addition, the roles of microRNAs (miRNAs) and epigenetic regulation such as DNA methylation and histone modification have received increasing attention in recent years [1]. The complex regulatory networks involving these mechanisms have been studied extensively in embryonic stem (ES) and induced pluripotent stem (iPS) cells and have revealed that the regulatory activity, in combination with transcription factors, is associated with pluripotency [2].

We previously assessed the expression pattern of miRNAs in human and mouse ES and iPS cells [3]. We found that several miRNAs were highly expressed in undifferentiated iPS cells [3]. Among these, we focused on miRNA- (miR-) 142-3p in the current study. miR-142 was first identified in hematopoietic cells [4], where it plays various roles in differentiation and functions during hemopoiesis [5–7]. miR-142 is highly conserved among vertebrates [8] and has been implicated in cardiac cell fate determination [9], osteoblast differentiation [10], and vascular development [11]. In cancer,* miR-142-3p* was identified at the breakpoint of a* MYC* translocation in B-cell leukemia [12] and was mutated in 20% of diffuse large B-cell lymphomas [13]. It is also critically involved in T-cell leukemogenesis [14] and the migration of hepatocellular carcinoma cells [15].

miRNAs are transcribed by RNA polymerase II [16], which involves various transcription factors. In hematopoietic cells, specifically, Spi1, Cebpb, Runx1, and LMO2 have all been reported to regulate miR-142 expression [17, 18]. However, these transcription factors are mostly hematopoietic cell-specific, suggesting that the expression of miR-142 in undifferentiated iPS cells involves regulation of other factors. In this study, we examined the roles of miR-142-3p in iPS cells and found that miR-142-3p might be involved in the proliferation of iPS cells and in maintaining their immaturity. Furthermore, miR-142-3p might also play roles in the mesodermal differentiation of iPS cells. Our data suggest roles for the methylation of CpG motifs in the 5′ genomic region of miR-142-3p in suppressing its expression in fibroblasts. Luciferase analysis of the isolated genomic region of miR-142-3p supports the idea that the expression of miR-142-3p in cells including fibroblasts and iPS is regulated, at least partially, by DNA methylation.

## 2. Materials and Methods

### 2.1. Cell Lines, 5-Aza-2′-deoxycytidine (5-Aza-dC) Treatment, and Transfection

3T3 cells were cultured in the DMEM (Nacalai Tesque) supplemented with 10% fetal bovine serum (GIBCO) and 0.5% penicillin/streptomycin (Nacalai Tesque). Preparation and culture of mouse embryonic fibroblast (MEF) and tail-tip fibroblasts (TTF) are described previously [3]. ICR mice were purchased from local dealers, and all experiments with animals were approved by the Animal Care Committee of the Institute of Medical Science at the University of Tokyo. Mouse iPS cell line, SP-iPS, was from B6 mouse MEF with infection of 4 factors (Sox2, Oct3/4, Klf4, and c-myc) by using retrovirus [19]. Culture of the iPS cells and formation of embryoid body (EB) is described previously [3]. For treatment of 5-aza-dC, cells were treated with final concentration of 5 or 10 *μ*M 5-aza-dC (SIGMA) or dimethyl sulfoxide (DMSO) for control samples 6 hours after the cells were plated, and cells were cultured for 3 days before analysis unless otherwise noted. For plasmid transfection, 3T3 cells were plated in a 24-well culture plate 1 day before transfection. Transfection of luciferase plasmid was done by using Gene Juice Transfection Reagent (Novagen). Briefly, Gene Juice Reagent (1.5 *μ*L), plasmid (0.25 *μ*g in 0.25 *μ*L for each plasmid), and Opti-MEM (Gibco-Life Technologies) were mixed and added to 3T3 cells. For plasmid transfection to iPS, electroporation was employed. iPS cells were dissociated into single cells by 0.05% trypsin-EDTA, washed with PBS, and resuspended in Opti-MEM. For each transfection, 1 × 10^6^ cells/30 *μ*L were gently mixed with 15 *μ*g of plasmid and placed in 2 mm gap electroporation cuvette (Nepa Gene Co., Ltd.). The cells were electroporated for two times at 175 V, 2 ms at 50 ms interval (CUY21 EDIT, Nepa Gene Co., Ltd). Immediately after electroporation, 1 mL of iPS culture medium was gently added to the cuvette, and cells were transferred and cultured on feeder cells in iPS medium. On the following day, the cells were dissociated and stained with SSEA-1 marker. Subsequently, the GFP + SSEA-1 + double positive cells from study or control group were sorted by FACS (MoFlo, DakoCytomation) and used for cell proliferation and colony formation assay.

### 2.2. RNA Extraction and Real-Time PCR for Quantification of miRNAs and mRNA

Total RNA was extracted using the Sepasol (Nacalai Tesque), and level of mature miRNAs was detected using TaqMan MicroRNA systems (Applied Biosystems) using primer specific for each mature miRNA supplied by Applied Biosystems using Light Cycler 1.5 (ROCHE). Briefly, a total of 500 ng RNA were reverse-transcribed with Taqman Reverse-Transcription PCR Kit with specific primer for miR-142-3p. Then, cDNA was mixed with TaqMan Universal Master Mix (Applied Biosystems) and was subjected for real-time PCR. Ct value was analyzed with SDS 2.4 and RQmanager 1.2.1 and quantitated using 2^−ΔΔCt^ method (Livak, 2001). All data were normalized to endogenous control, the U6 snRNA. Sequences of the primers are T/brachyury 5′-cacaccactgacgcacacggt-3′, 5′-atgaggaggctttgggccgt-3′, Gata4 5′-agccggtgggtgatccgaag-3′, 5′-agaaatcgtgcgggagggcg-3′, Fgf5 5′-gcagtccgagcaaccggaact-3′, and 5′-ggacttctgcgaggctgcga-3′. For quantification of mRNA, total RNA (1 *μ*g) from each sample was used to generate cDNA using ReverTra Ace qRT-PCR RT Kit (Toyobo). Then, cDNA was mixed with Sybr Green Master Mix (ROCHE) and was subjected for real-time PCR using Light Cycler 1.5 (ROCHE). Expression levels of mRNA were compared to known standard samples and normalized to GAPDH.

### 2.3. Isolation and Bisulfite Treatment of Genomic DNA

Genomic DNA was isolated from ~5 × 10^6^ cells using the QIAamp DNA Mini and Blood Mini kit (Qiagen). Genomic DNA (1 *μ*g) was subjected for bisulfite conversion using EpiTect Bisulfite (Qiagen). The converted DNA was further subjected to PCR for A-tailing procedure with HotStarTaq DNA Polymerase (Qiagen). Regions covering up to 700 bp upstream of the miR-142 seed sequence were amplified and were cloned into pGEM-T Easy Vector (Invitrogen). All positive clones were sequence and methylation results obtained were analyzed by Quantification Tool for Methylation Analysis (QUMA, http://quma.cdb.riken.jp) which was used for detection of CpG island methylation [20].

#### 2.3.1. DNA Construction

**Figure 1:**
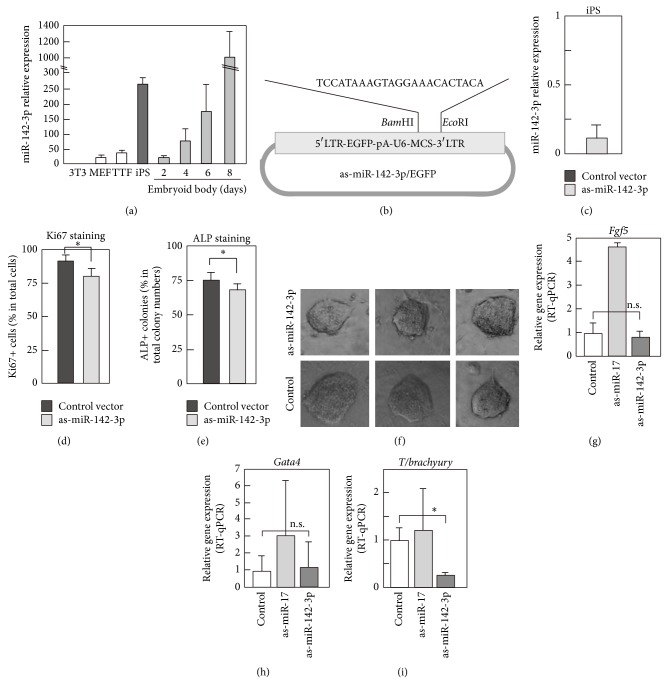
Differential expression level of miR-142-3p in fibroblasts and iPS. (a) Expression of miR-142-3p was examined by RT-qPCR in various cells. Total RNA was extracted from indicated cells, and RT-qPCR was done using TaqMan MicroRNA systems. U6 shRNA was used as a control. Experiments were done three times using independently prepared cells, and average values with standard deviation are shown. (b) Schematic representation of antisense- (as-) miR-142-3p and EGFP expression plasmid. LTR was used to drive EGFP, and U6 promoter was used to drive as-miR-142-3p. (c) Effect of overexpressed as-miR-142-3p for expression level of endogenous miR-142-3p in iPS cells. as-miR-142-3p/EGFP or control vector was transfected into iPS, and, after 24 hours, level of miR-142-3p in iPS was examined by RT-qPCR. Data were expressed as relative expression level of miR-142-3p in as-miR-142-3p/EGFP expressing cells to that in control vector expressing cells. Experiments were performed three times, and average values with standard deviation are shown. (d, e, and f) Effects of expression of as-miR-142-3p for proliferation and alkaline phosphatase (ALP) expression of iPS. as-miR-142-3p/EGFP plasmid was transfected into undifferentiated iPS, and EGFP positive cells were purified by a cell sorter. Then EGFP positive cells were cultured for 2 days for Ki67 immunostaining and for 5 days for ALP assay. Immunostaining with anti-Ki67 antibody or ALP staining was done, and positive cells were counted under a microscope. Experiments were performed three times, and average values with standard deviation are shown. In (f), morphology of representative colonies of as-miR-142-3p or control vector transfected iPS is shown. (g–i) Expression of lineage marker genes in embryoid body (EB). iPS cells were transfected with as-miR-142-3p/EGFP or as-miR-17/EGFP as a control, purified according to their expression of EGFP, and then subjected to an EB formation. After 6 days of culturing in EB formation condition, the differentiation of cells into the ectodermal (g), endodermal (h), and mesodermal (i) lineages was assessed using RT-qPCR with primers against* Fgf5*,* Gata4*, and* T brachyury*, respectively. *P* value, ∗ < 0.05 and n.s. > 0.05, was calculated by Student's *t*-test.

Plasmids containing antisense sequences of mature miR-142-3p or miR-17 expression plasmid were constructed as follows: double strand DNA, which encode antisense of mature miR-142-3p or miR-17, was inserted downstream of U6 promoter using* Bam*HI and* Eco*RI sites of pMX retrovirus vector containing EGFP after 5′ LTR (Figure 1(b)). Expression plasmids for mouse Oct4, Sox2, Klf, and Myc were purchased from AddGene.

#### 2.3.2. Cell Sorting, Cell Staining with Alkaline Phosphatase (ALP), and Immunostaining

Cells' sorting was done using a MoFlo (DakoCytomation). ALP staining was done using BCIP-NBT solution kit for alkaline phosphatase stain (Nacalai Tesque) according to the manufacturer's instructions. Immunostaining was done using antibody anti-Ki67 proliferation antigen (BD Biosciences), and the primary antibody was visualized using appropriate secondary antibody conjugated with Alexa 488 (Molecular Probes).

#### 2.3.3. Luciferase Analysis

3T3 cells were plated in a 24-well culture plate 1 day before transfection and transfected with luciferase plasmid (0.25 *μ*g) by using Gene Juice Transfection Reagent (Novagen). Six hours after transfection, cells were treated with final concentration of 10 *μ*M of 5-azacytidine and were cultured for 3 days. Cells were harvested using Cell Culture Lysis Reagent 5X (Promega). Luciferase activity toward a luciferase assay substrate (Promega) was measured with a luminometer (Lumat LB9507, Berthold Technologies).

## 3. Results

### 3.1. Characterization of miR-142-3p Expression in iPS Cells, Embryoid Bodies, and Fibroblasts

We previously characterized the expression pattern of miRNAs in mouse and human iPS and ES cells using miRNA arrays and found that miR-142-3p, but not miR-142-5p, was expressed at high levels in iPS cells (see Supplementary Figure 1 available online at http://dx.doi.org/10.1155/2014/101349) [3]. We first confirmed the expression pattern of miR-142-3p using quantitative reverse transcription-polymerase chain reaction (qRT-PCR). miR-142-3p was expressed at a high level in undifferentiated iPS cells, whereas fibroblasts such as 3T3, mouse embryonic fibroblasts (MEFs), and tail-tip fibroblasts (TTF) expressed only very low levels (Figure 1(a)). When iPS cells were differentiated by formation of embryoid bodies (EBs), the expression of miR-142-3p fell to very low levels on day 2 but then increased on the following days (Figure 1(a)).

#### 3.1.1. Functional Analyses of miR-142-3p in iPS Cell Physiology

We next constructed an expression plasmid encoding antisense miR-142-3p (as-miR-142-3p) and enhanced green fluorescent protein (EGFP; Figure 1(b)). A plasmid without insertion of antisense miR-142-3p was used as a control for all experiments. The effect of expressing as-miR-142-3p on endogenous miR-142-3p was then examined and confirmed in mouse iPS cells (Figure 1(c)). Specifically, as-miR-142-3p/EGFP was transfected into undifferentiated iPS to analyze the role of miR-142-3p in the proliferation and maintenance of immaturity in iPS cells. Twenty-four hours after transfection, EGFP-positive cells were purified using a cell sorter and cultured for 3 days. Cell proliferation was then assessed by immunostaining for Ki67, a proliferative marker (Figure 1(d)). The population of Ki67-positive cells was slightly, but significantly, lower in as-miR-142-3p-expressing iPS cells (Figure 1(d)). We then counted the number of alkaline phosphatase- (ALP-) positive iPS colonies, and significantly fewer ALP-positive cells were found within the as-miR-142-3p-expressing iPS colonies (Figure 1(e)). Morphology of colonies of iPS cell was indistinguishable between control and as-miR-142-3p expressing samples (Figure 1(f)).

We then analyzed the roles of miRNA-142-3p on the ability of iPS cells to differentiate. iPS cells were transfected with as-miR-142-3p/EGFP, purified according to their expression of EGFP, and then subjected to an EB formation assay. An expression plasmid containing antisense sequence against miR-17, which is expressed at very high levels in undifferentiated iPS cells [3, 21], was used as a control. After 6 days, the differentiation of cells into the ectodermal, endodermal, and mesodermal lineages was assessed using real-time quantitative PCR (qPCR) with primers against* Fgf5*,* Gata4*, and* T brachyury*, respectively (Figures 1(g), 1(h), and 1(i)). Data revealed that as-miR-142-3p, but not as-miR-17, suppressed the expression of* T brachyury*, which is expressed specifically in cells of the mesodermal lineage [22] (Figure 1(i)). The expression of as-miR-142-3p did not affect the expression of* Fgf5* or* Gata4*, although as-miR-17 enhanced expression of* Fgf5*, as expected (Figures 1(g) and 1(h)).

### 3.2. 5-Aza-2′-deoxycytidine Treatment Upregulates miR-142-3p in Fibroblasts

**Figure 2:**
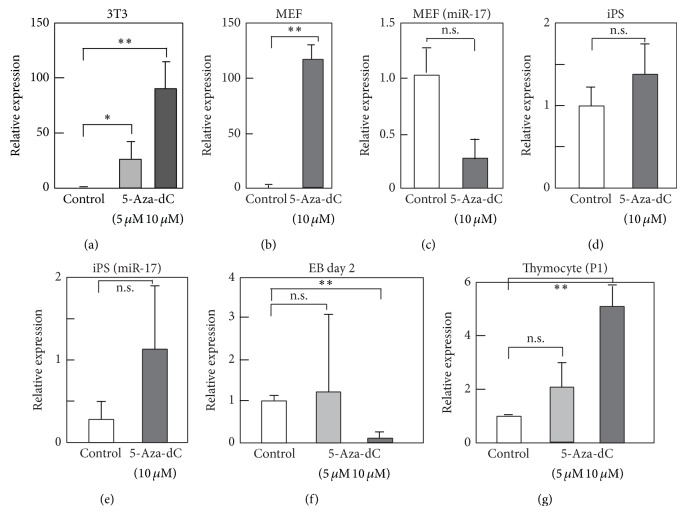
5-Aza-2′-deoxycytidine (5-aza-dC) treatment upregulates miR-142-3p in fibroblasts. (a–g) 3T3 (a), MEF (b, c), iPS (d, e), embryoid body (EB) formed from mouse iPS (f), or mouse thymocytes (g) were treated with 5-aza-dC at indicated final concentration (5 or 10 *μ*M). Cells were cultured for 3 days in the presence of 5-aza-dC, except for EB, which was treated with 5-aza-dC for two days. Control cells were treated with DMSO. Then, cells were harvested, and total RNA was extracted. Level of miR-142-3p or miR-17 was examined by RT-qPCR. Value of U6 was used as a control. Values are expressed as relative to those of control samples of each cell type and are average of 3 or 4 times experiments with standard deviation. *P* value, ∗∗ < 0.01, 0.01 <  ∗ < 0.05, and n.s. > 0.05, was calculated by Student's *t*-test.

To assess the transcriptional regulation of miR-142-3p expression, we examined its 5′ genomic sequence and identified 25 CpG motifs in a region covering ~1000 base pairs (bp) upstream of the miR-142-5p core sequence (Supplementary Figure 2). We hypothesized that miR-142-3p expression is regulated epigenetically by DNA methylation in iPS cells and fibroblasts. MEFs and 3T3 cells were treated for 3 days with 5 or 10 *μ*M of 5-aza-2′-deoxycytidine (5-aza-dC), a DNA methyltransferase inhibitor (Dnmt), and the levels of miR-142-3p were assessed using real-time qPCR. The expression of miR-142-3p was upregulated by 5-aza-dC treatment (Figures 2(a) and 2(b)). In contrast, the levels of miR-17 were rather reduced but not significantly by 5-aza-dC (Figure 2(c)), whereas the expression of neither miR-142-3p nor miR-17 was changed significantly by 5-aza-dC in undifferentiated iPS cells (Figures 2(d) and 2(e)). We also examined the effects of 5-aza-dC on miR-142-3p in EBs and found that 10 *μ*M 5-aza-dC rather suppressed the expression (Figure 2(f)). We also examined the effects of 5-aza-dC for miR-142-3p expression in thymocytes. Levels of miR-142-3p were upregulated slightly by 10 *μ*M of 5-aza-dC, but to a much lesser extent than observed in fibroblasts (Figure 2(g)). Taken together, these results suggest that miR-142-3p is suppressed by DNA methylation in fibroblasts but that the downregulation of miR-142-3p during EB formation might be regulated by a different mechanism.

### 3.3. Proximal CpGs in the miR-142-3p Genomic Region Regulate Transcriptional Activity

**Figure 3:**
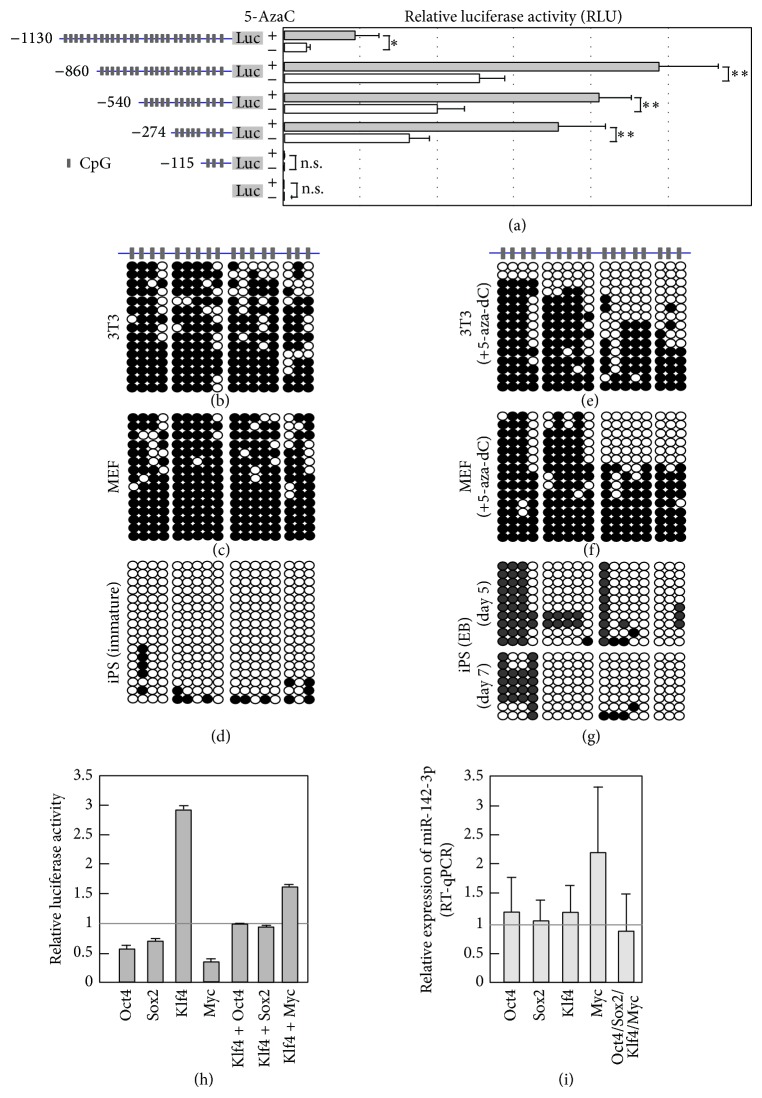
Expression of miR-142-3p was regulated by DNA methylation. (a) Left panel shows schematic representation of luciferase constructs. Luciferase analysis using plasmids containing indicated length fragments of the 5′ upstream region of miR-142-3p-luciferase was done. Plasmid was transfected into 3T3 cells, and, after 6 hours, samples were treated with DMSO or 5-aza-dC (10 *μ*M) and cultured for additional 3 days. Then cells were harvested, and luciferase activities were examined. Values are average of 3 times independent experiments with standard deviation. *P* value, ∗∗ < 0.01 and n.s. > 0.05, was calculated by Student's *t*-test. (b–g) CpG methylation of 5′ upstream region of miR-142-3p was examined by bisulfite conversions. Genomic DNAs extracted from 3T3, MEF in the presence or absence of 5-aza-dC, iPS, or EB prepared from iPS were subjected to bisulfite sequence. 5-Aza-dC was present in the culture medium of 3T3 or MEF 72 hours before harvesting cells for genomic DNA extraction (e, f). (h) 3T3 cells were transfected with expression plasmid of Oct4, Sox2, Klf4, or Myc with −540 Luc. For control sample, empty expression plasmid and −540 Luc were transfected. Cells were harvested after 3 days of culture, and luciferase analysis was conducted. (i) 3T3 cells were transfected with indicated expression plasmid, and, after 3 days, cells were harvested, and total RNA was extracted. Expression level of endogenous miR-142-3p was examined by RT-qPCR. (h, i) Values are relative to control vector transfected samples and average of 4 independent samples with SD.

We next performed promoter analyses of different fragments of the 5′ upstream region of miR-142-3p using luciferase assays. Previous reports indicated that transiently transfected plasmids could be CpG-methylated in the cells* de novo* [23, 24]. Luciferase constructs were transfected into 3T3 cells, which were cultured in the presence or absence of 5-aza-dC for 3 days. Luciferase assays were then performed. In the absence of 5-aza-dC, the −274, −540, and −860 Luc constructs showed significant luciferase activity, which increased gradually when longer promoters were used (Figure 3(a)). In contrast, −1130 Luc had very low luciferase activity, suggesting the presence of a region between −860 and −1130 nucleotides (nt) that inhibited luciferase activity. When cells were cultured in the presence of 5-aza-dC, the luciferase activity of −274 Luc was upregulated significantly (Figure 3(a)). Since there are six CpGs in the region covering −274 to ATG, we speculated that the methylation status of the proximal six CpGs might play roles in the upregulation of luciferase activity.

### 3.4. CpG Methylation in the 5′ Genomic Region of miR-142-3p

To further elucidate the role of CpG sites and DNA methylation in regulating the expression of miR-142-3p, we analyzed the methylation status of the CpG sites identified in the region up to 700 bp upstream of the pre-miR-142-5p core region (Supplementary Figure 2) using bisulfite conversion. Analyses performed in 3T3 cells and MEFs revealed that the CpG sites were hypermethylated (Figures 3(b) and 3(c)). In contrast, those in undifferentiated iPS cells were hypomethylated (Figure 3(d)). We then analyzed the effects of 5-aza-dC on the methylation status in 3T3 cells and MEFs. Treatment with 5-aza-dC lowered methylation levels significantly, particularly at the proximal eight CpGs (Figures 3(e) and 3(f)). CpGs were also hypomethylated in day 5 EBs (Figure 3(g)), even though the expression of miR-142-3p was much lower than in undifferentiated iPS cells (Figure 1(a)).

### 3.5. Roles of Pluripotency-Related Transcription Factors in miR-142-3p Gene Activation

We next investigated the possible involvement of the pluripotency-associated transcription factors Oct4, Sox2, Klf4, and c-Myc in the regulation of miR-142-3p transcription. The miR-142-3p promoter-luciferase construct (−540 Luc) was transfected into 3T3 cells with one of the four transcription factors, and luciferase assays were performed 3 days later. Luciferase activity was strongly upregulated by Klf4, whereas the other three transcription factors suppressed luciferase activity (Figure 3(h)). In addition, cotransfection with Klf4 and one of Oct4, Sox2, and c-Myc lowered luciferase activity compared with Klf4 alone (Figure 3(h)). We then analyzed the effects of overexpressing these transcription factors on the expression of endogenous miR-142-3p in 3T3 cells, but no effects were observed (Figure 3(i)).

## 4. Discussion

This study revealed that miR-142-3p is expressed in undifferentiated iPS cells, but not in fibroblasts, and DNA methylation might play a pivotal role in suppressing miR-142-3p expression in fibroblasts. Previous studies revealed that the transcription of miRNAs could be regulated by DNA methylation [25, 26]. miR-142-3p was reported to be upregulated in the human melanoma cell line WM1552C after treatment with 5-aza-dC [27], suggesting that the expression of miR-142-3p was attenuated by DNA methylation not only in fibroblasts, but also in melanocyte lineage cells. In the current study, 5-aza-dC did not enhance the expression of miR-142-3p in mouse P1 thymocytes, supporting the hypothesis that DNA methylation is not a major mechanism that regulates the expression of miR-142-3p in hematopoietic cells.

The expression of miR-142-3p in hematopoietic cells is regulated by various transcription factors that also play important roles in hematopoiesis [17, 18]. The sequence of pre-miR-142 is highly conserved among vertebrates [8]. In addition, the expression of human miR-142 was recently reported to be regulated by the methylation of a CpG in its enhancer region in mesenchymal cells [8]. Although no similarity was found in the mouse and human upstream genomic regions (~2000 nt) of miR-142-3p, miR-142 expression is regulated by CpG methylation in both species.

Methylation changes occur predominantly at the end of reprogramming. The genomic region harboring pluripotency-associated genes including Nanog, Oct4, and Zfp42 is demethylated very late during reprogramming [28]. When 5-aza-dC is present during this period, an increased number of embryonic stem cell-like colonies are observed [29]. Furthermore, 5-aza-dC enhances the generation of iPS cells by inhibiting Dnmt1 activity [30]. The expression of miR-142-3p might be desilenced by the suppression of DNA demethylation and stimulated by other genes that play roles in the late phase of reprogramming. We observed that Klf4 upregulated luciferase activity but that Klf4 did not enhance the expression of endogenous miR-142-3p in 3T3 cells. Therefore, we hypothesize that a molecular environment related to reprogramming, which 3T3 cells lack, might be required for miR-142-3p expression. We identified several potential binding sites for c-Myc and Sox2 in the genomic region up to 1 kb from the miR-142 mature sequence using the Genomatix Software Suite (http://www.genomatix.de/solutions/genomatix-software-suite.html). Therefore, the combination of these transcription factors in a wider genomic region might cooperate for the full induction of miR-142-3p expression.

TGF-*β*R1 and TGF-*β*R2 were both predicted to be targets of miR-142-3p [31], and TGF-*β*R1 was identified as a direct target in non-small-cell lung cancer [32]. TGF-*β*1 is involved in the reprogramming process in which the inhibition of TGF-*β* signaling enhances the efficiency of reprogramming [33]. More recently, a report indicated that the miR-142-3p-mediated regulation of Wnt signaling could modulate the proliferation of mesenchymal progenitors [34]. The identification of miR-142-3p target genes in the TGF-*β* and Wnt signaling pathways further supports the hypothesis that miR-142-3p is involved in the regulation of iPS cell physiology.

## 5. Conclusions

miR-142-3p, which is highly expressed in iPS cells but not in fibroblasts, plays roles in the proliferation and differentiation of iPS cells. The expression of miR-142-3p is suppressed by DNA methylation of its CpG motifs in the 5′ genomic region in fibroblasts.

## Supplementary Material

Supplemental Fig. 1: miR142-5p was strongly expressed in all the examined mouse iPS cell lines when cells keep immature state.
Supplemental Fig. 2: Region covering 1 kb of 5' upstream genomic region of miR142 contains number of CpG sites.
