# Supplementary material for: DNA Methylation Is Involved in the Expression of miR-142-3p in Fibroblasts and Induced Pluripotent Stem Cells
Source: Stem Cells Int. 2014 Dec 2;2014:101349. doi: 10.1155/2014/101349 (PMC4269320; doi:10.1155/2014/101349)
Supplement: Supplementary file 1 — Supplemental Fig. 1: miR142-5p was strongly expressed in all the examined mouse iPS cell lines when cells keep immature state. Supplemental Fig. 2: Region covering 1 kb of 5' upstream genomic region of miR142 contains number of CpG sites. [file 101349.f1.pdf]

Supplemental Figure 1 Siti et al.

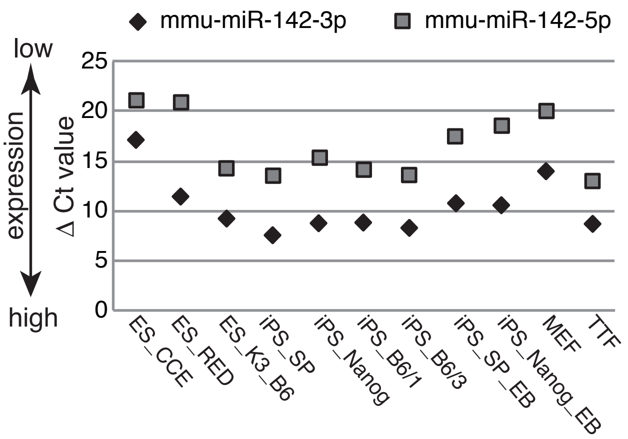

Expression of miR142-5p and miR-142-3p

Expression level of miR142-5p and miR142-3p was originally examined by microRNA array and published in Siti et al., Plos One, 2013. Results of miR-142-5p and miR142-3p were extracted from Table S4 of Siti et al. ΔCt value was calculated by subtracting Ct values for mammalian U6 snRNA, which was selected as an internal control, from Ct value of each sample.

Supplemental Figure 2 Siti et al.

-1130
CAGCAGGAGGGGCTGGGTGCCAGAGCTCCCTAACAAAGTTTTAGGCTTAG
GAGCAATGACTCTTGGATCCACTAGCCTTCACTGGGGACAGCTTTTACAA
TCTTTTAAATCTTCTTGGGTTTTTTGTTTTTTGTTTGTTTGTTTGTTTTT
TGGGGGAAGCTGGGAACCGAACCCAGGGCTTTGCGCTTACCATTGAGCTA
AATCCCCAACCCCATCTTTTAAATCTACAGAATTAAAAAAGGAAAAATTA
GCCGGGCGGTGGTGGCGAACACCTTTAATCCCTGCACTCGGGAGGCAGAG
-860
ACAGATGGATTTCTGAGTTCGAGGCCAGCTTGGTCTACAAAGTGAGTTCT
AGGACAGCTTGGGCTATACAGAGAAACCCTGTCTCGAAAAACCAAAAAAA
AAAAAAAAAAAAAAAAAAAGGAAAAATTGGCTTCTGAGCCCCCCTCTCGA
AAATAACAGTCTCTAGCTCTTAGGGAGGAGGGCCAGAGAGTAGGGAGGAG
TTGCGAGAGTGGGAGAGTGGGCTGAAACAGTGAGACTGGTATCGGCAAGT
GGTGCCCAGGCATTTTTTCCACGCAACATGGCCGCCATTTCTGCCAACAC
-540
ACTTGGATTCCAGCTTGAGGAGCAGGAATGAGGGAGTCAGGGGCCTTGTT
CCAATGGGGAGACTAGAGACAGAGGCGTGTGGGAGTGGAGGCCTTTGTCT
AATGGGAAGGCTGGGGACCGAGGCCTTTATGGGAGGAGGCCATGGCTGCC
TCCGTTGGATTCAAGACTGTGGGTCATACGGCTGGGAAGCACCACCCAGG
ATGTTGCAGTTCTAAAGATGGGGATGAGCCTTCTGGAGTGGCTGTGGTCT
-274
TTACCTGAGTGTCCCCGAGAAGGGAGAGAATAGGGTGGAAACCCGGAGGG
GGTGGGGAGTGGAGGGAAGAAGGTTACAAAGAGGAAGTTGGGCGGGGGAA
GAAGAGGCTCATCTGGCGCCATGTTGAGTCACCACCCACAAGGCCCAGGG
-115
CGGGCCCTCTAGGGGGCCACAGGCAGGGTGGAGCGGTCCCTGGGAAGTTA
CACGGAGGGGAGGGGGCAGGGGCCTTTATTAAAGCAGGAGTCGGCAAGAA
AAGCAGGTGGCCTGAAGAATCCCCGTGGACAGACAGACAGTGCAGTCACC
CATAAAGTAGAAAGCACTACTAACAGCACTGGAGGGTGTAGTGTTTCCTA
CTTTATGGATGAGTGCACTGTGGGCTT

CpG analyzed by bisulfite assay, CpG not analyzed by bisulfite assay

miR-142-5p core sequence, miR-142-3p core sequence

Supplemental Fig. 1

5' up-stream genomic sequence of miR142

Sequence of 5' genomic region of miR-142 retrieved from Ensemble (http;//asia/ensembl.org/Mus_musculus/Gene/). Arrows and number indicate genomic region contained in luciferase constructs.
